# Impedance Analysis and Noise Measurements on Multi Walled Carbon Nanotube Networks

**DOI:** 10.3390/ma14247509

**Published:** 2021-12-07

**Authors:** Usha Philipose, Yan Jiang, Brianna Western, Michael Harcrow, Chris Littler, Ashok Sood, John W. Zeller, Bobby Lineberry, A. J. Syllaios

**Affiliations:** 1 Department of Physics, University of North Texas, Denton, TX 75077, USA; yanjiang@my.unt.edu (Y.J.); brianna.western@unt.edu (B.W.); michael.harcrow@unt.edu (M.H.); chris.littler@unt.edu (C.L.); aj.syllaios@unt.edu (A.J.S.); 2 Magnolia Optical Technologies, Inc., Woburn, MA 01801, USA; aksood@magnoliaoptical.com (A.S.); jwzeller@magnoliaoptical.com (J.W.Z.); 3 USARMY Combat Capabilities Command (DEVCOM) Technology Development Directorate, Building 7612, Redstone Arsenal, AL 35898, USA; bobby.i.lineberry.civ@mail.mil

**Keywords:** carbon nanotubes, multi-walled, impedance, permittivity, 1/f noise, packing density, tunneling, Nyquist analysis

## Abstract

The electrical impedance characteristics of multi-walled carbon nanotube (MWCNTs) networks were studied as a function of CNT concentrations in the frequency range of 1 kHz–1 MHz. The novelty of this study is that the MWCNTs were not embedded in any polymer matrix and so the response of the device to electrical measurements are attributed to the CNTs in the network without any contribution from a polymer host matrix. Devices with low MWCNT packing density (0.31–0.85 µg/cm^2^) exhibit a frequency independent plateau in the low-frequency regime. At higher frequencies, the AC conductivity of these devices increases following a power law, characteristic of the universal dynamic response (UDR) phenomenon. On the other hand, devices with high MWCNT concentrations (>1.0 µg/cm^2^) exhibit frequency independent conductivity over the entire frequency range (up to 1 MHz), indicating that conduction in these devices is due to direct contact between the CNTs in the network. A simple single-relaxation time electrical equivalent circuit with an effective resistance and capacitance is used to describe the device performance. The electrical noise measurements on devices with different MWCNT packing densities exhibit bias-dependent low-frequency 1/f noise, attributed to resistance fluctuations.

## 1. Introduction

With growing demands for miniaturization and for small electronic devices with many integrated functionalities, there have been systematic studies on several nanoscale systems, including research on conducting thin films of carbon nanotubes (CNTs) [1,2]. At the nanoscale level, the properties of materials are significantly influenced by surface and quantum effects and there are theoretical works that predict the mechanical, chemical and electrical behavior of materials at the nanoscale [3,4].

An important area of CNT application is that of bolometric detection of near- and mid-infrared (IR) radiation. High infrared bolometric photoresponse has been observed in multiwall carbon nanotube (MWCNT) films at room temperature [5,6]. Due to their excellent electrical and mechanical properties another promising application has been the integration of CNTs into micro-electromechanical system (MEMS) devices [7,8]. In several applications, CNTs incorporated into polymer matrices have resulted in nano-composites with enhanced mechanical, electrical and sensing properties [9,10,11,12]. This is why most of the research has focused on CNT-based nano-composites where, at a critical volume fraction (percolation threshold), there is a steep increase in electrical conductivity. The aspect ratio and intrinsic properties (metallic/semiconducting) of the CNTs play a significant role in determining the percolation threshold [13,14]. In this context, multi-walled CNTs (MWCNTs) are particularly favored since, while single-walled CNTs (SWCNTs) can exhibit both semiconducting and metallic properties, MWCNTs are mostly metallic in character with conductivities in the range of 10^5^–10^7^ S/m [15,16].

There have been several reports on the electrical properties of nano-composites comprising of CNTs, mainly on the determination of their direct current (DC) electrical properties [10,13,17,18]. However, there are a very limited number of articles available on the alternating current (AC) and dielectric behavior of such nano-composites [15,19,20,21,22,23,24] and, in all of these works, the CNTs are embedded into a polymer matrix. The thickness and type of the polymer affects the tunneling resistance and strongly influences the conduction mechanism. 

The novelty of this work is that the study focuses on the electrical behavior of a CNT network that is not embedded in any polymer matrix; thereby attributing all measured electrical response to the behavior of the CNTs in the network without any contribution from a host (polymer) matrix. The study uses electrical impedance spectroscopy to determine the frequency response of the resistive and capacitive elements of the nanotube network. To the best of our knowledge, the use of impedance spectroscopy to study conduction mechanisms in CNT networks not embedded in a polymer matrix is quite rare. The quantitative description of the alternating current (AC) conductivity features is analyzed to reflect properties of the underlying nanotube network with low and high CNT concentrations. Knowing how the electrical properties of a network of randomly aligned clusters of CNTs respond to AC bias is of particular importance when designing devices, particularly for sensing applications, particularly in the design of uncooled bolometers where impedance influences both temperature coefficient of resistance (TCR) and noise, two important parameters that influence bolometer performance.

## 2. Materials and Methods

The MWCNTs used in this work were purchased from Nano-Integris (batch no. MW35-056, 0.1 g/100 mL) and had diameters in the range of 30–40 nm, which includes the thickness of the surfactant shell of sodium dodecyl-sulfate (SDS). The MWCNTs were metallic in nature, as verified by back-gate measurements. This paper follows up on our previous work [25], where the meniscus dragging dispersion (MDD) technique was used to disperse the MWCNTs on an Si/SiO_2_ substrate [26]. In order to ensure a good interface between the CNTs and between the metal contact and CNT, the surfactant shell was removed following the procedure described in our earlier work [25]. The fabricated devices had MWCNT concentrations ranging from 0.3 µg/cm^2^ to about 1.6 µg/cm^2^ and their AC response was tested using the Z–theta module of an Agilent Semiconductor Device Analyzer (B1500A). The contact pad patterns (2 microns apart) for the electrical leads were generated by photo-lithography on the Si/SiO_2_ substrate and 50 nm of gold (Au) was deposited on the contact area by thermal evaporation. The experiments were performed at room temperature and at 0–1 V bias. The amplitude of the sinusoidal voltage excitation was maintained at 30 mV for all devices, with frequencies ranging from 1.0 kHz to 1.0 MHz. Direct measurements of the amplitude and the phase shift of the resulting current allows for the determination of the real and imaginary components of the complex impedance. Following this, the real and imaginary components of dielectric permittivity, and AC conductivity were determined for all six devices.

Noise measurements were made at room temperature with devices mounted in a cryostat to ensure that all measurements were made under dark conditions. A Keithley 428 source was used to bias the device and also functioned as a low noise current amplifier. The gain for each device was adjusted by a variable feedback resistor within the pre-amplifier circuit. The amplified signal was converted to a noise voltage–frequency spectrum by an HP 35670A dynamic signal analyzer (DSA).

### Modelling the MWCNT Network

**Figure 1:**
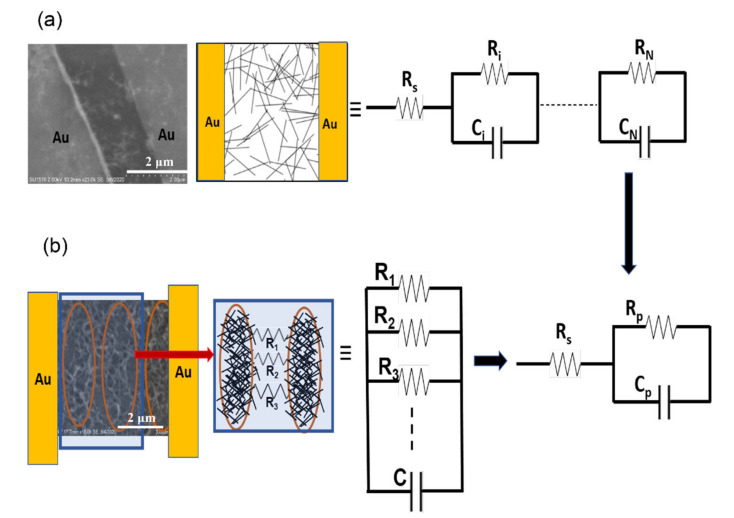
Device schematic showing the MWCNTs contacted by two Au electrodes and the equivalent circuit representing the conducting network. (**a**) Randomly oriented homogeneous distribution of MWCNTs; (**b**) Clustering of MWCNTs in the network due to high packing density.

The dispersion of the MWCNTs in the devices results in networks with different degrees of homogeneity. Since the MWCNTs are metallic, their intrinsic resistance is negligible compared to the tunneling contact between them. There is also a capacitive contribution arising from charge buildup on CNTs separated by a dielectric medium. A simplified model is shown in Figure 1a, where the CNTs in the network are randomly oriented but uniformly dispersed (low packing density devices). In this case, the network can be modelled as a set of parallel RC-circuits connected in series [15]. Assuming the resistance and capacitance of the *i*th MWCNT in the network to be Ri and Ci, respectively, there will be N such parallel units in the network. 

The total impedance of ‘N’ number of such series-connected elements is expressed as [15]:

(1) $$Z=R_{s}+\overset{N}{\underset{i=1}{\sum }}\frac{R_{i}}{1+j\omega R_{i}C_{i}}$$

where ω=2πf is the angular frequency. The resistance *R_s_* is associated with contact resistance and includes resistance between the metal pads and the MWCNTs that are in contact with it. In the case of high packing density devices, the dispersion of the MWCNTs in the network is not homogeneous. Therefore, each device can be treated as having CNTs in small clusters (highly conducting islands of CNTs) and electrical transport occurs between neighboring clusters by electron tunneling between them with negligible capacitive contributions. Figure 1b is an SEM image and a schematic of one such device that shows small clusters of CNTs connected to each other through a few conducting arms of individual CNTs that assist with electron tunneling [15]. The conducting arms are represented by resistors *R*_1_, *R*_2_, … in parallel (Figure 1b). The capacitive contribution in this case is from sufficiently separated clusters that does not permit efficient tunneling. The total device resistance is a sum of the equivalent resistances R and capacitances C, defined by a simple parallel resistance and capacitance *R_p_* and *C_p_* model. In both cases described in Figure 1, the randomly dispersed MWCNTs in the network can be modelled by a single relaxation time model with relaxation time, $\tau_{0}$ defined as $\tau_{0}=R_{p}C_{p}$. Following the impedance analysis described by Helseth [15], the real and imaginary parts of impedance can be expressed as [15]:

(2) $$Re\left[Z\left(\omega \right)\right]=Z^{\prime }=R_{s}+\frac{R_{p}}{1+{\left(\omega \tau_{0}\right)}^{2}}$$

and

(3) $$Im\left[Z\left(\omega \right)\right]=Z^{\prime \prime }=\frac{-\omega \tau_{0}R_{p}}{1+{\left(\omega \tau_{0}\right)}^{2}}$$

where the real component ($Z^{\prime }$) represents the network resistance, and the imaginary part ($Z^{\prime \prime }$) represents the reactance (loss factor). The relationship between the real and imaginary components (Equations (2) and (3)) depicts a semicircle of diameter $R_{p}$ in the $Z^{\prime \prime }$–$Z^{\prime }$ plane and can be used to determine the modeling parameters $R_{s}$, $R_{p}$ and $C_{p}$.

## 3. Results and Discussion

In our previous work [25], the DC resistance of a thin MWCNT network was shown to scale with the areal mass density of MWCNTs by a power law, with a percolation exponent of 1.42 and a percolation threshold of 0.12 µg/cm^2^. All devices used in this study have concentrations beyond the percolation threshold.

**Figure 2:**
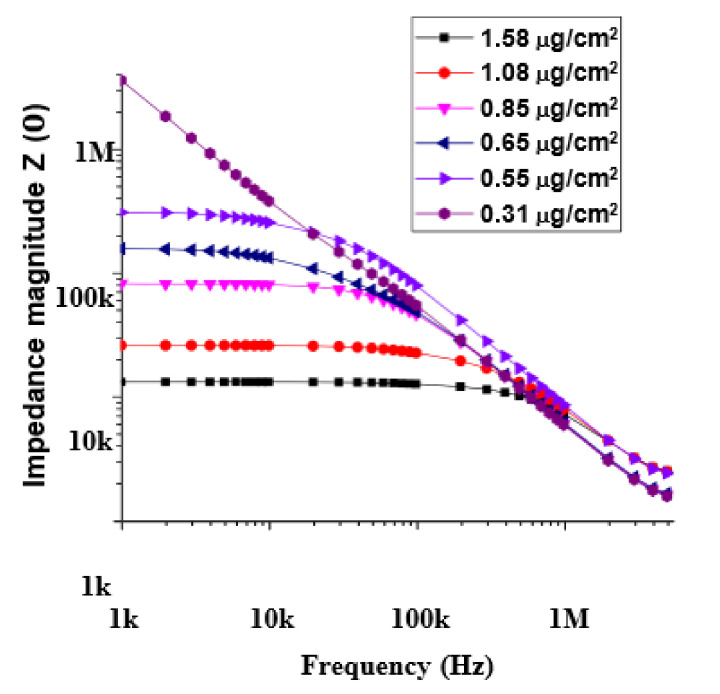
Magnitude of impedance versus frequency for MWCNT devices fabricated on SiO_2_/Si substrate. The packing densities of MWCNTs in the devices are shown in the corresponding legend and varied from about 0.3 µg/cm^2^ to about 1.5 µg/cm^2^.

Figure 2 depicts the magnitude of impedance as a function of frequency, for the six devices with different MWCNT concentrations and DC network resistances. In the low-frequency regime, samples with relatively low MWCNT concentrations (in the range of 0.3 µg/cm^2^ to about 0.7 µg/cm^2^) show high impedance values (greater than 100’s of kΩ). Networks with higher CNT concentrations show significantly lower impedance values (less than 100 kΩ). The formation of conducting paths within the disordered network determines the electrical conductivity, that has both DC (resistive) and AC components (capacitive). The resistive component depends mainly on the number of contacts between the dispersed MWCNTs and is independent of frequency, while the capacitive component is influenced by capacitive interactions between adjacent MWCNTs and is therefore influenced by the frequency of the applied field. We also repeated these measurements on glass substrates with matching packing densities and found they had comparable resistive (DC) component of conductivity. This indicates that the frequency-independent region (resistive component) is not influenced by the choice of substrate; instead, it is determined by the packing density of the CNTs and the number of contacts in the disordered network. 

Figure 2 also depicts a shift from a frequency independent regime (DC) to a frequency dependent regime (AC) occurring at a characteristic frequency ($f_{c}$) that is dependent on the packing density of MWCNTs in the device. The transition is not evident at the low packing density of 0.31 µg/cm^2^ and is most likely because the transition occurs at a frequency below 1 KHz and is outside the scope of our current experimental set up. At each packing density, the magnitude of the impedance remains fairly constant in the low- frequency ranges and declines rapidly when the frequency exceeds $f_{c}$. Geng et al. [22] defines $f_{c}$ as the point between the low- and high-frequency ranges, where the plot of impedance magnitude shows an inflection and at which the phase angle Φ=π/4. For frequencies below $f_{c}$, the resistive component of the network dominates the overall impedance and hence its magnitude is independent of frequency. For frequencies greater than $f_{c}$, the capacitive effect of the network becomes dominant and the magnitude of the impedance decreases. At some frequency much higher than $f_{c}$, the impedance magnitude value is expected to tend to zero. The value of $f_{c}$ and the magnitude of the impedance below $f_{c}$ are dependent on the packing density of CNTs in the network. At all frequencies exceeding $f_{c}$, the magnitude of the impedance has a power law dependence on the test frequency, defined as [22]:

(4) $$\left|Z\left(f\right)\right|\propto f^{n}$$

where the exponent n ranges from 0.68 to 0.98. The limited exponent range (Table 1) agrees well with the AC universality of disordered solids [22,27].

For the device with no measurable DC conductivity (very low packing density) the value of the exponent was close to 1.0. The plot of Figure 2 also shows that, for all devices (packing densities higher than the percolation threshold) at frequencies above 10^5^ Hz, the $\left|Z\left(f\right)\right|$ values merge. This has been attributed to the presence of a space charge polarization effect at low frequencies; the effect becoming negligible at higher frequencies [28]. 

**Figure 3:**
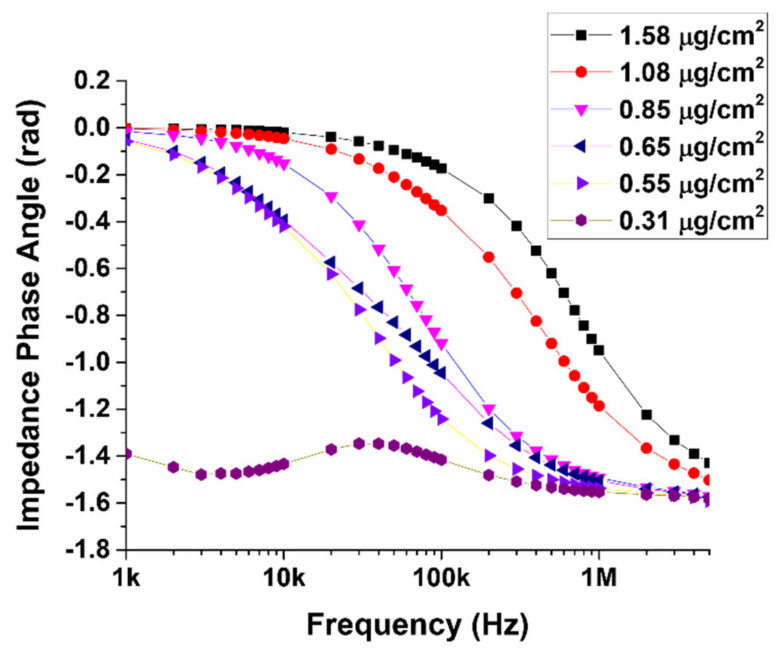
Phase plots for the six MWCNT devices with different packing densities fabricated on SiO_2_/Si substrate.

Figure 3 shows the variation of phase angle with frequency for the six devices. The imaginary part of the impedance represents the reactance (loss factor) and is related to the capacitive behavior of the device. As seen in the plot of Figure 3, devices with high MWCNT content showed no peaks (loss peak or relaxation peak), whereas a single peak is observed at the low MWCNT concentration of 0.31 µg/cm^2^. The absence of loss peaks at higher packing densities indicates that there are no relaxation processes at these concentrations in the measured frequency range.

**Figure 4:**
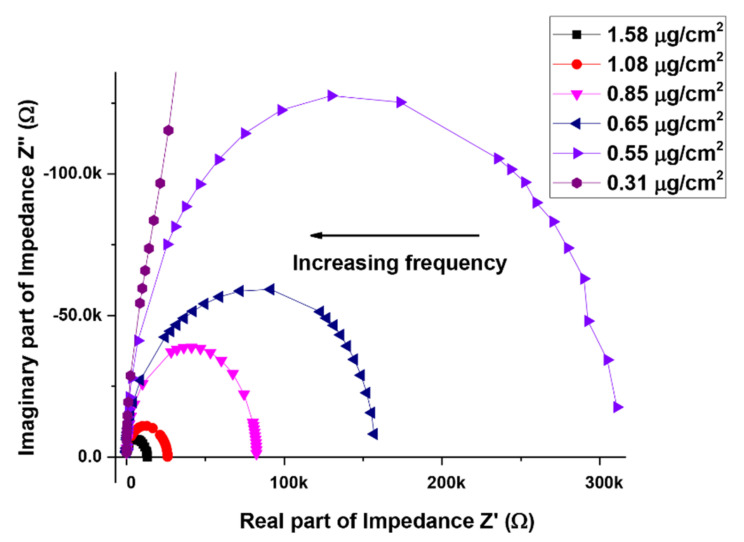
Variation of real and imaginary parts of impedance for the six fabricated devices with different packing density of MWCNTs.

**Figure 5:**
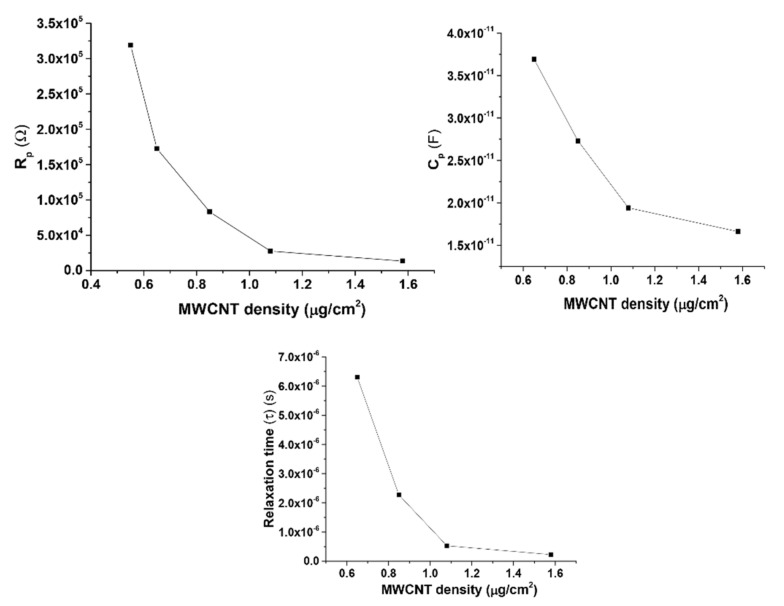
Dependence of $R_{p}$, $C_{p}$ and τ on packing density of MWCNTs, values obtained from the single relaxation time equivalent circuit model applied to the impedance data.

To study the electrical properties of the devices in greater detail, the real and imaginary parts of the complex impedance ($Z^{\prime }$ and $Z^{\prime \prime }$) are plotted on a normal scale (Nyquist plot), shown in Figure 4. The plot shows a semicircular arc for each device (not within measurable range for the lowest density device) and is characterized by smaller diameter arcs for high packing density devices. The intercept of the semicircles on the real axis (diameter of the semicircle) corresponds to the device resistance. The angular frequency $\omega_{max}$ of the peak maxima is related to the reciprocal of the relaxation time τ [28]. The series resistance $R_{s}$ depends on the concentration of MWCNTs in the device. Devices with largest density of MWCNTs had R_s_ in the range of 100 s of ohms. This value increased to KΩ for the low-density devices. Figure 5 shows the extracted values of $R_{p}$, $C_{p}$ and τ as a function of MWCNT density. The value for $R_{p}$ decreases by an order of magnitude as the CNT density doubles. This is attributed to the formation of clusters in high packing density devices. On the other hand, the change in $C_{p}$ is not that significant and is of the order of tens of pF. The relaxation time (τ) decreases by almost an order of magnitude as the packing density doubles and then remains fairly constant.

The response of the disordered network of MWCNTs to an applied AC electric field is best described by the real part of the complex electrical conductivity (usually called AC conductivity $\sigma_{AC}$). At low frequencies, the direct contact between CNTs in the network results in the formation of a continuous “percolation” path between the electrodes, resulting in a frequency independent DC conductivity ($\sigma_{DC}$). As the frequency increases, $\sigma_{AC}$ exhibits dispersion and increases in a power law fashion. This behavior, referred to as the universal dynamic response (UDR) is evident in the low packing density device and is defined by the equation [21,29]:

(5) $$\sigma_{AC}=\sigma_{DC}+A\omega^{s}$$

**Figure 6:**
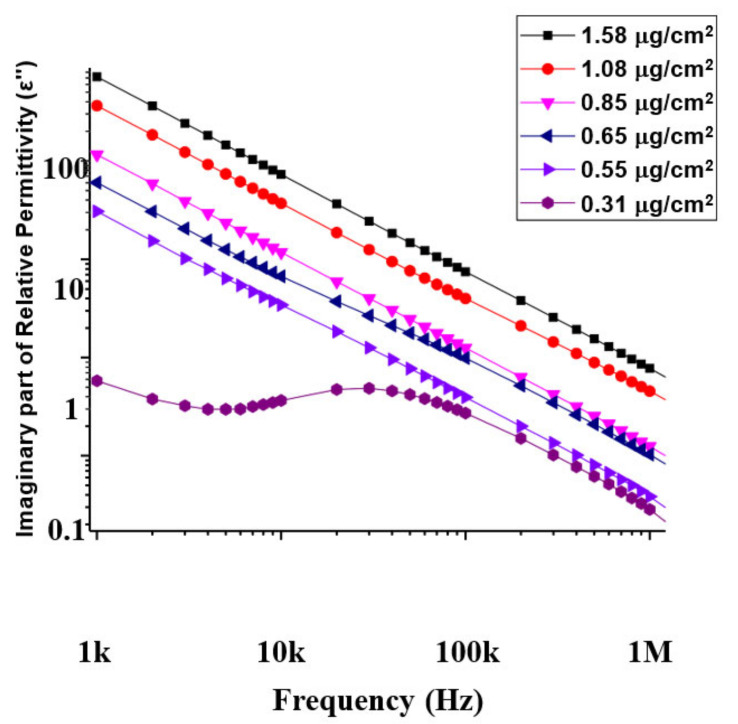
Variation of imaginary part of relative permittivity with frequency for the six devices.

The first term in Equation (5) represents the frequency independent conductivity ($\sigma_{DC}$), attributed to the direct contact between the MWCNTs. The second term in Equation (5) ($A\omega^{s}$) represents the frequency-dependent component of AC conductivity. In this term, A is a temperature-dependent constant, ω is the angular frequency, and s is a temperature and frequency-dependent exponent with values 0 < s < 1. The quantity $A\omega^{s}$ is associated with conductivity resulting from electrons tunneling and hopping between MWCNTs in the network. The increase in conductivity at high frequencies has also been attributed to the removal of any charge build up as a result of space charge polarization [30]. To calculate $\sigma_{AC}$, the imaginary part of complex relative permittivity ($\varepsilon^{\prime \prime }$) is used, defined by the equation [30]:

(6) $$\varepsilon^{\prime \prime }=\frac{Z^{\prime }}{\omega C_{0}Z^{2}}$$

where $C_{0}$ is the geometric capacitance $=\epsilon_{o}A/d$, $\epsilon_{o}$ is the permittivity of vacuum, A is the area and d is the network thickness (assumed to be thickness of the MWCNTs, ≈40 nm). The dependence of $\varepsilon^{\prime \prime }$ on frequency and MWCNT content (Figure 6) can be analyzed to draw conclusions about the polarizability and dielectric loss (conductance) of the CNT network [31,32]. Based on the dielectric analysis, the AC conductivity ($\sigma_{AC}$) can be calculated using the equation [30]:

(7) $$\sigma_{AC}=\omega \varepsilon_{0}\varepsilon^{\prime \prime }$$

**Figure 7:**
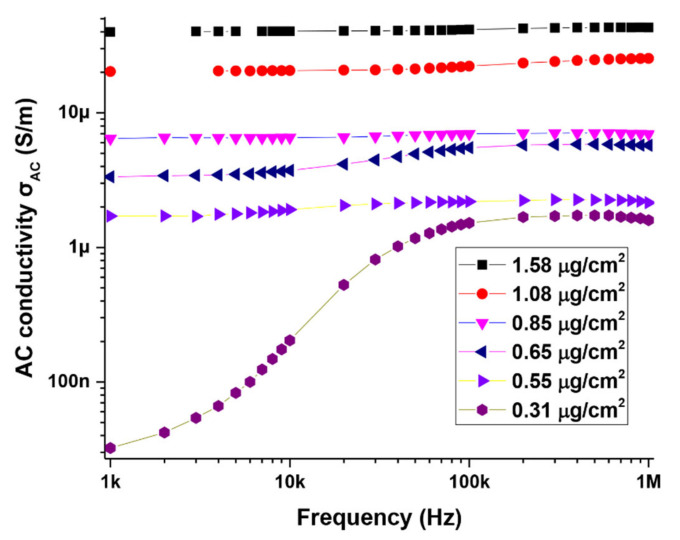
AC conductivity of MWCNT network as a function of frequency and MWCNT content.

Figure 7 is a plot of the ac conductivity of the MWCNT devices as a function of frequency and CNT concentration. On a log scale (Figure 7), only the low packing density device (0.31 µg/cm^2^) is seen to clearly exhibit typical UDR behavior that is characterized by a flat DC plateau in the low-frequency region, followed by a region of increased conductivity based on the power relation $A\omega^{s}$. The exponent s in this case was estimated to be 0.6. The lowest frequency in our current experimental set up is 1 kHz and so the flat DC portion of the curve for this device is not very evident, possibly occurring at frequencies below 1 kHz. The plot shows that for this device, $\sigma_{AC}$ increases and the increase will continue as long as the frequency of the field is lower than the maximum hopping frequency [27]. At larger field frequencies, $\sigma_{AC}$ stabilizes and becomes constant. A careful analysis of the plots for the higher packing densities (Figure 7) on a linear scale shows that devices with MWCNT densities of 0.55 µg/cm^2^, 0.65 µg/cm^2^ and 0.85 µg/cm^2^ also exhibit UDR behavior with exponents in the range of 0.5–0.7. The high packing density devices (1.08 and 1.58 µg/cm^2^) shows orders of magnitude increase in $\sigma_{DC}$, associated with an increase in connected conductive paths within the network as the CNTs get more densely packed.

**Figure 8:**
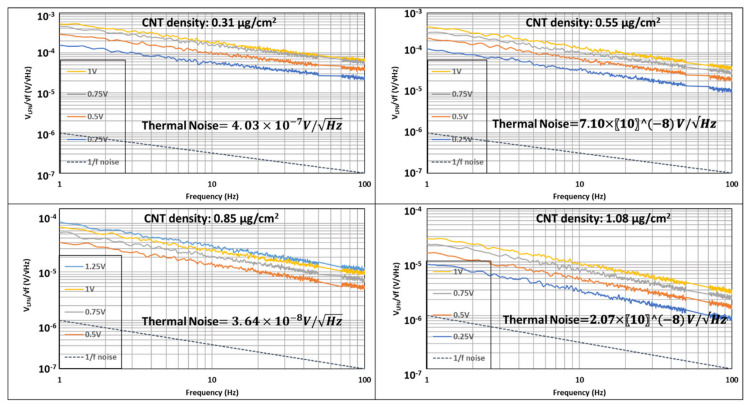
The 1/f noise amplitude spectral density as a function of bias voltage for devices with different packing density of CNTs.

Studies to analyze the signal to noise ratios of CNT networks are important for their potential use in electrical applications. Our results so far show that electrical transport through the network occurs by tunneling and the device resistance is related to the junction resistance between CNTs in the percolated network. Noise measurements were made on four devices with different packing densities. The noise characterization was performed by biasing the devices at four different DC bias values (in the range of 0.25–1.0 V) and measuring the spectral density of low frequency fluctuations with a spectrum analyzer. The noise spectra were acquired at room temperature in the 1–100 Hz frequency range. Figure 8 depicts the noise power at different bias values measured across each device.

At zero bias, the noise was flat and agreed with the thermal Nyquist level $S_{V}=4k_{B}TR$. At finite bias levels, excess noise is measured. The amplitude of the excess noise is defined by the equation: [33]

(8) $$S_{V}=\frac{V_{1/f}^{2}}{\Delta f}=\frac{AV_{Bias}^{2}}{f^{\gamma }}$$

where $A=\alpha_{H}/n$. $\alpha_{H}$ is the Hooge constant, and n is the number of charge carriers. After subtraction of the thermal baseline, the excess noise was found to vary as 1/f. The dashed line in each of the plots is a 1/f noise line. For all devices, the exponent γ was found to range between 1.05 ± 0.02, revealing a 1/f behavior induced by resistance fluctuations [34]. Due to limitations in our experimental setup, we were unable to perform noise measurements at kHz frequency. However, extrapolation of the data shown in Figure 8 shows that at high frequencies of the order of few kHz, the 1/f noise amplitude will approach or become lower than the thermal noise.

**Figure 9:**
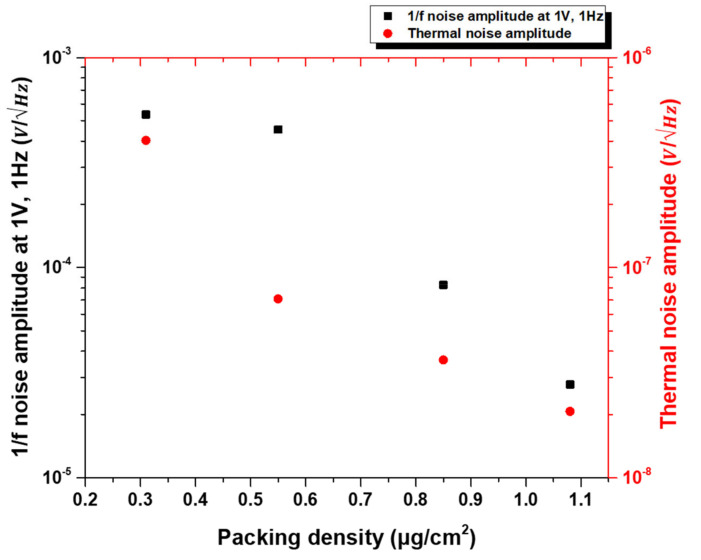
Dependence of 1/f noise amplitude (at 1 V, 1 Hz) and thermal noise on packing density of CNTs in the network.

The dependence of the amplitude of 1/f noise (at 1 V bias and at the low frequency of 1 Hz) and thermal noise on the packing density of CNTs in the network is shown in Figure 9. As seen in the plot, the high packing density devices (low resistance devices) have orders of magnitude lower 1/f noise. It is also evident that, as the packing density of CNTs increases, the value of 1/f noise approaches the thermal noise $4k_{B}TR$. These results support our model, that for the high packing density devices there are a number of parallel paths through which current flows through the network. Hence, the independent fluctuations in different paths tend to cancel, thereby reducing the fluctuations in the total resistance or 1/f noise. The noise amplitude increases in low packing density devices where the connectivity between the CNTs is lower. This is explained on the basis of a reduced current flow through a smaller number of paths, which increases resistance fluctuations and 1/f noise amplitude.

## 4. Conclusions

The impedance spectra of a network of MWCNTs are investigated as a function of the concentration of CNTs in the device. The experimental data compare well with the existing literature on CNT-based composites and can be explained in terms of a simple electrical RC-circuit with a single relaxation time, indicating that the polymer matrix in previously reported works does not influence the basic characteristics of the device. In this work, the contact reactance was assumed to be negligible for the low- and high-packing density devices. This needs further validation and the role played by the contact reactance at different frequencies should be analyzed and incorporated into a revised R-C model of the CNT network. At a low packing density of CNTs, the frequency dependent conductivity is most likely due to electron tunneling in addition to direct contact between the MWCNTs in the network. At high packing densities, direct contact between CNTs in the network is responsible for the frequency independent conduction. A characteristic frequency $f_{c}$, dependent on the packing density of CNTs in the network was identified for each device. At frequencies higher than $f_{c}$, the magnitude of the impedance was found to have a power law dependence on the test frequency, where the limited exponent range agrees with the AC universality of disordered solids. Low-frequency voltage-noise-spectral measurements on devices with different packing density of CNTs shows that the excess noise is 1/f -like in nature and follows the Hooge model.

## Figures and Tables

**Table 1:** Exponent n, calculated from Equation (4) for different packing density devices fabricated on SiO_2_/Si.

CNT Concentration (µg/cm^2^)	n
1.58	0.68
1.08	0.73
0.85	0.80
0.65	0.77
0.55	0.78
0.31	0.98

## Data Availability

The data presented in this study are available on request from the corresponding author.
